# Attenuation of Zinc Finger Nuclease Toxicity by Small-Molecule Regulation of Protein Levels

**DOI:** 10.1371/journal.pgen.1000376

**Published:** 2009-02-13

**Authors:** Shondra M. Pruett-Miller, David W. Reading, Shaina N. Porter, Matthew H. Porteus

**Affiliations:** 1 Department of Pediatrics, University of Texas Southwestern Medical Center, Dallas, Texas, United States of America; 2 Department of Biochemistry, University of Texas Southwestern Medical Center, Dallas, Texas, United States of America; Stanford University School of Medicine, United States of America

## Abstract

Zinc finger nucleases (ZFNs) have been used successfully to create genome-specific double-strand breaks and thereby stimulate gene targeting by several thousand fold. ZFNs are chimeric proteins composed of a specific DNA-binding domain linked to a non-specific DNA-cleavage domain. By changing key residues in the recognition helix of the specific DNA-binding domain, one can alter the ZFN binding specificity and thereby change the sequence to which a ZFN pair is being targeted. For these and other reasons, ZFNs are being pursued as reagents for genome modification, including use in gene therapy. In order for ZFNs to reach their full potential, it is important to attenuate the cytotoxic effects currently associated with many ZFNs. Here, we evaluate two potential strategies for reducing toxicity by regulating protein levels. Both strategies involve creating ZFNs with shortened half-lives and then regulating protein level with small molecules. First, we destabilize ZFNs by linking a ubiquitin moiety to the N-terminus and regulate ZFN levels using a proteasome inhibitor. Second, we destabilize ZFNs by linking a modified destabilizing FKBP12 domain to the N-terminus and regulate ZFN levels by using a small molecule that blocks the destabilization effect of the N-terminal domain. We show that by regulating protein levels, we can maintain high rates of ZFN-mediated gene targeting while reducing ZFN toxicity.

## Author Summary

Zinc finger nucleases (ZFNs) are a powerful tool to create site-specific genomic modifications in a wide variety of cell types and organisms and are about to enter human gene therapy clinical trials. An important aspect of using ZFNs for use in gene therapy is to minimize off-target effects. We made ZFNs that contain destabilizing domains on their amino-terminus. The expression level of the modified ZFNs could be increased transiently by the addition of a small molecule, either a proteasome inhibitor or Shield1. We demonstrate that off-target effects can be reduced without compromising gene targeting efficiency by using small molecules to limit the maximal expression of the ZFNs to a narrow window. The ability to regulate ZFN expression using small molecules provides a new strategy to minimizing off-target effects of ZFNs and may be an important way of ultimately using ZFNs for clinical use in gene therapy protocols.

## Introduction

Homologous recombination is a natural mechanism that cells use for a variety of processes including double strand break (DSB) repair [1]. To repair a DSB by homologous recombination, the cell usually uses the sister chromatid as a donor-template but can use other pieces of DNA such as extrachromosomal DNA. Gene targeting uses homologous recombination to make a precise genomic change and is commonly used experimentally in a variety of cells including yeast and murine embryonic stem cells. However, the spontaneous rate of homologous recombination is too low in mammalian somatic cells (10^−6^) to be commonly used experimentally or therapeutically [2]–[5]. The rate of gene targeting, however, can be increased (to over 10^−2^) by creating a gene specific DSB [2], [6]–[10].

Zinc Finger Nucleases (ZFNs) can create site-specific DSBs and have been shown to increase the rate of gene targeting by over 5 orders of magnitude [11]–[13]. ZFNs are chimeric proteins that consist of a specific DNA binding domain made up of tandem zinc finger binding motifs fused to a non-specifc cleavage domain from the FokI restriction endonuclease (the development of which is reviewed in [14]). By changing key residues in the DNA binding domain, ZFN binding specificity can be altered providing a generalized strategy for delivering a site-specific DSB. However, many ZFNs have been shown to have cytotoxic effects [2], [15]–[17]. Several studies suggest that this toxicity is caused by “off-target” DSBs. For example, a zinc finger protein containing no nuclease domain was not toxic when transfected into HEK293 cells (unpublished data). Similarly, Beumer et al. (2006) have shown that ZFNs containing point mutations to inactivate the nuclease domain do not exhibit cytotoxicity in flies [18]. There have been two published strategies for reducing the number of “off-target” breaks: (1) increase the specificity of the ZFN by protein engineering or (2) force heterodimerization of the ZFN pairs [16], [19]–[24]. Here, we explore a third strategy to reduce cytotoxicity by small molecule regulation of ZFN protein levels.

By creating ZFNs from zinc finger DNA binding domains that are more specific, toxicity is reduced. While on-target cutting is generated by heterodimerization of a ZFN pair at its target site (at least 18 base pairs), off-target cutting can be mediated by either homodimer pairs or heterodimer pairs. Modifications in the nuclease to prevent homodimerization results in ZFNs with reduced toxicity [16],[20],[21]. We found, however, that this reduction can come at a cost of reduced activity in stimulating gene targeting [16] (Wilson et al., manuscript submitted).

It has been shown that the rate of gene targeting can be increased, up to a point, by increasing the amount of transfected ZFN expression plasmids [16],[18]. However, very high levels of ZFN expression cause toxicity without increasing targeting rates [16],[18]. These observation lead to the hypothesis that reduced toxicity could also be obtained by being able to regulate ZFN expression. This “Goldilocks” phenomenon means that being able to titrate the amount of ZFN protein is critical to optimizing ZFN mediated gene targeting.

Porteus and Baltimore demonstrated that maximal DSB-mediated gene targeting occurs within 60 hours of transfection of DNA [2]. Expression of ZFNs outside this window will increase toxicity without increasing targeting. We hypothesized that if we could narrow the time of ZFN protein expression, we could reduce toxicity while maintaining high rates of targeting. In this study, we use two previously described strategies to regulate protein levels and apply them to ZFNs. We show that by regulating protein levels, we reduce the number of “off-target” DSBs and reduce toxicity, while maintaining high ZFN-stimulated gene targeting activity.

## Results

### Ubiquitin Tagging and the N-End Rule as a Strategy to Regulate ZFN Protein Levels

The ability to regulate ZFN protein levels could theoretically give optimal rates of gene targeting with minimal toxicity. Degradation signals or “degrons” are specific domains that confer instability on a protein [25]. The N-end rule correlates the *in vivo* half-life of a protein to the N-terminal amino acid; some residues are destabilizing while other residues are stabilizing [26],[27]. Normal N-terminal processing precludes simply adding a desired residue to the N-terminus of a protein. By adding a ubiquitin moiety (Ub) to the N-terminus of a protein, the N-terminal amino acid of a protein can be controlled. In eukaryotes, the Ub-X-POI (where POI is protein of interest) fusion is cleaved by Ub-specific processing proteases immediately before X (where X is an amino acid residue) [28]. This cleavage leaves the X residue as the N-terminal amino acid and thus affects protein stability. It has been established by several groups that an N-terminal arginine is a degradation signal [26],[28].

It is also possible to create poorly cleavable or uncleavable Ub-X-POI fusions. If the ubiquitin protein is not cleaved from the POI, the protein can undergo ubiquitin fusion degradation [29]. That is, the Ub-X-POI fusion can be further ubiquitinated and thereby “marked” for degradation by the proteasome. This allows for another strategy to create short-lived POIs. By substituting the last residue of the ubiquitin moiety from glycine to valine and using a valine linker (Ub-VV-POI), the ubiquitin moiety can no longer be cleaved from the POI [28].

**Figure 1:**
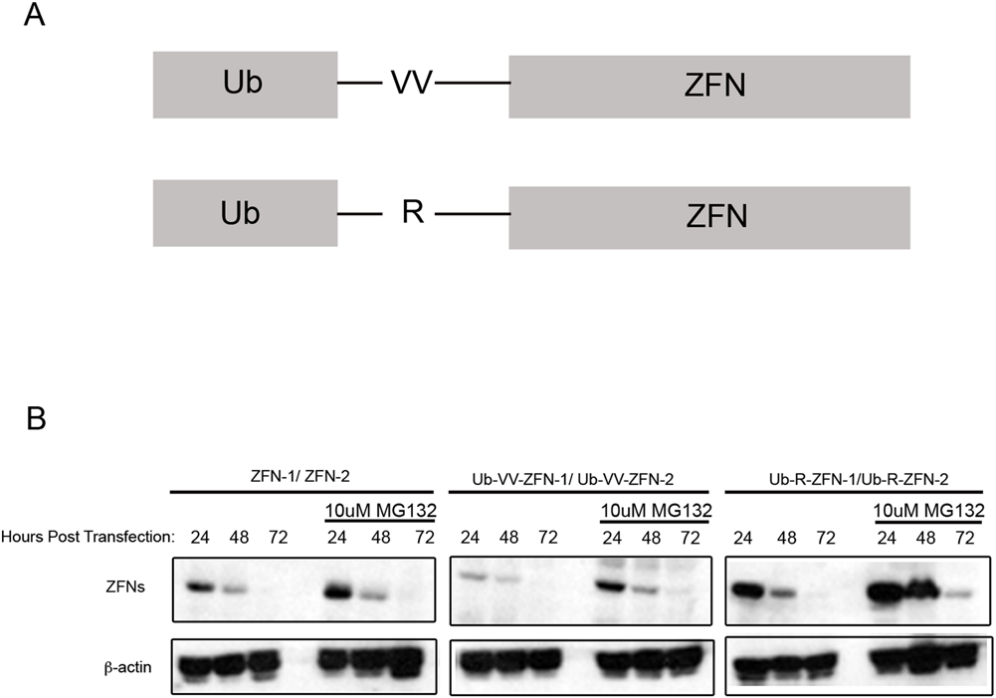
Characterization of Ub-X-ZFNs that display drug-dependent stability. (A) Genetic fusion of a ubiquitin moiety to a ZFN with either a “VV” linker or an “R” linker. (B) Expression profile of unmodified and Ub-X-ZFN proteins in the presence and absence of 10 uM of the proteasome inhibitor MG132 from 18–22 hours post-transfection. *HEK*293FT cells were transiently transfected with vectors encoding either ZFN-1/ZFN-2, Ub-VV-ZFN-1/-2, or UB-R-ZFN-1/ZFN-2. ZFNs were detected using Western blot analysis with an anti-Flag antibody. ZFN-1/ZFN-2 and Ub-R-ZFN-1/ZFN-2 were approximately 37 kD and Ub-VV-ZFN-1/ZFN-2 were approximately 47 kD. The size difference between the Ub-X-ZFNs is due to the Ub-moiety being cleaved off when linked via an R-linker. β-actin serves as a loading control.

We created a pair of Ub-VV-ZFNs and Ub-R-ZFNs fusion proteins (Figure 1A) to destabilize a pair of previously validated ZFNs targeting the GFP gene that contained the wildtype *FokI* domain [16]. The Ub-VV-ZFNs were made to take advantage of the potential destabilizing effect of a covalently linked N-terminal ubiquitin, and the Ub-R-ZFNs were made to take advantage of the potential destabilizing effect of an N-terminal arginine. Expression of the ZFN chimeras in transiently transfected HEK293 cells was examined by Western blot analysis (Figure 1B). The size of the Ub-VV-ZFNs corresponded with the expected size of an uncleaved fusion protein. The size of the Ub-R-ZFNs corresponded with the size of the unmodified ZFNs, confirming that the ubiquitin moiety was cleaved.

The addition of the proteasome inhibitor MG132 can increase the levels of Ub-X-POI fusion proteins [28]. We therefore examined the expression of ubiquitin linked ZFNs and unmodified ZFNs in the presence and absence of MG132 (Figure 1B). The addition of the proteasome inhibitor had little effect on the unmodified ZFNs. In contrast, addition of MG132 to cells transfected with ubiquitin modified ZFNs produced a striking increase in the expression relative to the level of expression of the modified proteins in the absence of MG132. It is interesting to note that even the untreated Ub-R-ZFNs had an increase in protein levels relative to the unmodified ZFNs (Figure 1B, compare Ub-R-ZFNs, +MG132 at 24 hours to unmodified ZFNs at 24 hours). Although expression of Ub-R-ZFNs is higher than the unmodified ZFNs, the UB-R-ZFNs levels decrease more rapidly suggesting that the N-terminus arginine is, in fact, destabilizing. We hypothesize that the addition of the ubiquitin moiety to the N-terminus aided in protein folding of the ZFNs and thus produced higher expression.

**Figure 2:**
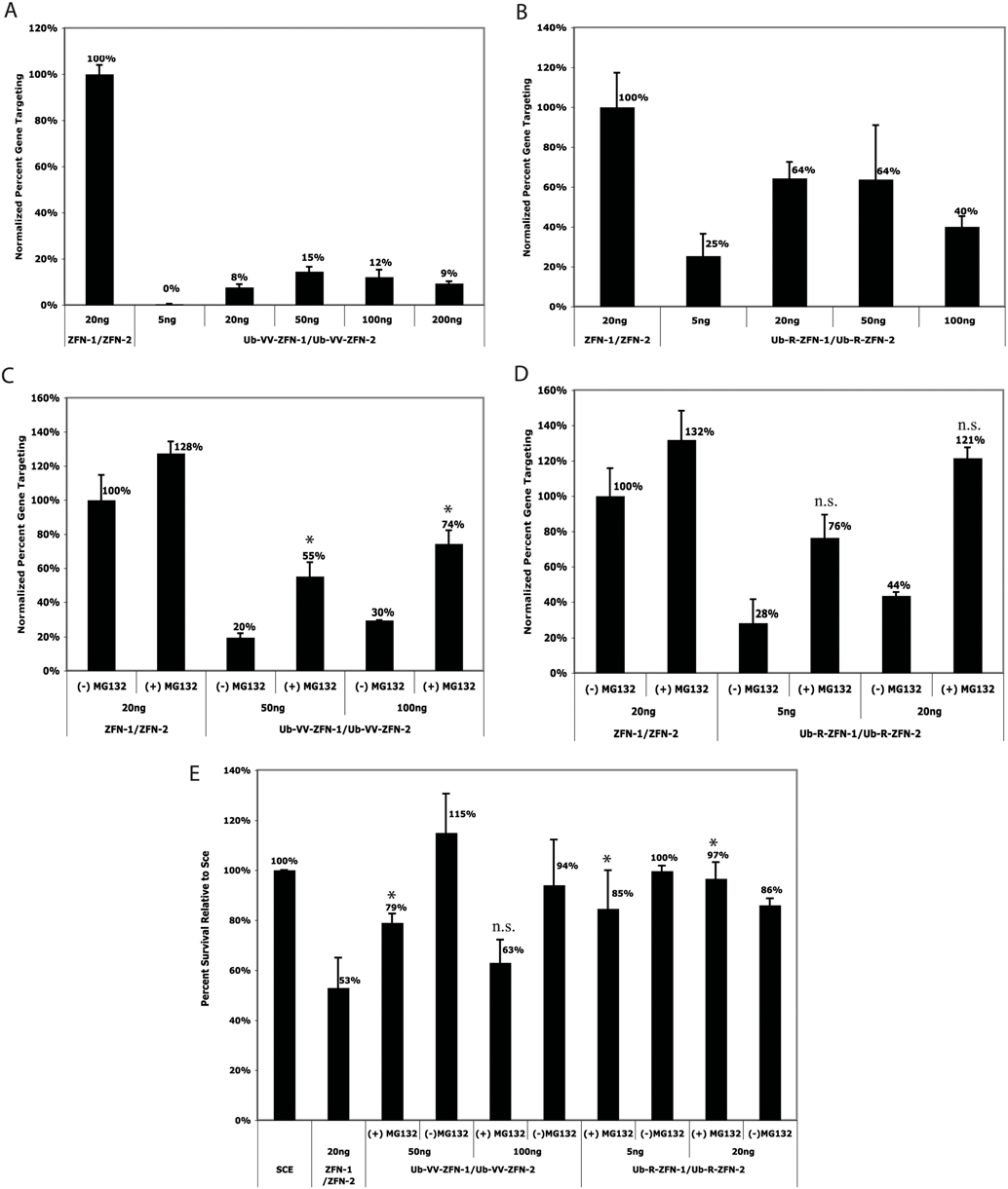
Analysis of Ub-X-ZFNs. Unless otherwise indicated, rates of gene targeting at day 3 were normalized to the rate of gene targeting achieved using 20 ng of the unmodified ZFNs without drug treatment as this was previously determined to be the conditions used to obtain optimal gene targeting with the unmodified ZFNs. The absolute rate of gene targeting using ZFN-1/ZFN-2 at 20 ng in *HEK*293 cells was about 20,000 GFP positive cells per million cells transfected (about 2%). (A) Titration of transfected DNA of Ub-VV-ZFNs in the gene targeting assay with increasing amounts of DNA. (B) Titration of transfected DNA of Ub-R-ZFNs in the gene targeting assay with increasing amounts of DNA. (C and D) Gene targeting in the absence and presence of 10 uM MG132 for given ZFN pairs at stated DNA concentrations. (E) Toxicity assay for all iterations of Ub-modified and unmodified ZFNs tested in the gene targeting assay relative to Sce. A value of <100% indicates decreased cell survival as compared with Sce, and demonstrates a toxic effect. Statistical analysis was performed using the Student's T-test comparing ZFN-1/ZFN-2 at 20 ng with no drug treatment to Ub-modified ZFNs treated with MG132. “*” indicates a P-value of <.05 and “n.s.” indicates no statistical significance or a P-value of >.05. Error bars are the standard deviation for three samples.

We compared the activity of the ubiquitin linked ZFNs to the unmodified ZFNs using a GFP gene targeting assay. In this assay, gene targeting is measured by the correction of a chromosomally integrated mutated GFP target gene [2]. We normalized the gene targeting rate for each condition to the rate obtained for the optimal amount of the unmodified ZFNs as previously determined [16]. We first compared activities of the Ub-VV-ZFNs and Ub-R-ZFNs with increasing amounts of DNA in the absence of proteasome inhibitor to that of the unmodified proteins (Figure 2A and 2B). In the absence of drug, both the Ub-VV-ZFNs and the Ub-R-ZFNs, at all DNA concentrations tested, produced lower amounts of gene targeting compared to rates produced using the unmodified pair. The Ub-VV-ZFNs produced lower rates of gene targeting in the absence of drug compared to the Ub-R-ZFNs.

We next evaluated the rate of gene targeting produced by the Ub-modified proteins in the presence of MG132 compared to when the drug was absent (Figure 2C and 2D). Both the Ub-VV-ZFNs and Ub-R-ZFNs produced increased rates of gene targeting in the presence of MG132 compared to when the drug was absent. The rate of gene targeting produced by the Ub-VV-ZFNs in the presence of drug was not as high, however, as rates produced by the unmodified protein. In contrast, in the presence of drug, the Ub-R-ZFNs produced equivalent rates of gene targeting as compared to the unmodified proteins.

In order to determine if the ubiquitin modification of these ZFNs reduced the cytotoxicity associated with unmodified ZFNs, we used a flow cytometry based cell survival assay (the “toxicity assay”) [16]. In this assay, we use a non-toxic endonuclease, *I-SceI* (hereafter called Sce), as the standard for a non-toxic nuclease to which we normalize relative amounts of toxicity. The percent of surviving cells transfected with a potentially toxic nuclease is compared to the percent of surviving cells transfected with Sce. A lower percent of surviving cells is a sign of greater toxicity. As shown in Figure 2E, the percent survival relative to Sce of the unmodified ZFNs is about 50%. Both the Ub-VV-ZFNs and the Ub-R-ZFNs examined in this experiment produced lower toxicity and therefore a higher percentage of survival compared to the unmodified proteins. At 20 nanograms (ng) of Ub-R-ZFNs in the presence of drug, there was no observable toxicity in this assay. This is also the amount at which equivalent rates of gene targeting were obtained relative to the unmodified proteins (Figure 2D). In summary, we found that the VV-linked versions minimized toxicity at the cost of reduced targeting efficiency. In contrast, using the R-linked versions, we could decrease toxicity without losing targeting efficiency.

### The Destabilization Domain Method as a Strategy to Regulate ZFN Protein Levels

An alternative strategy to using ubiquitin involves linking a destabilization domain to the POI. This destabilization domain was engineered by making mutants of the FKBP12 protein, which is constitutively and rapidly degraded in mammalian cells [30]. Fusion of this destabilization domain to another protein confers instability to the fusion protein. In order to stabilize the protein, Banaszynski et al. (2006), developed a synthetic ligand (called Shield1) that binds the destabilization domain and protects the fusion protein from degradation [30].

**Figure 3:**
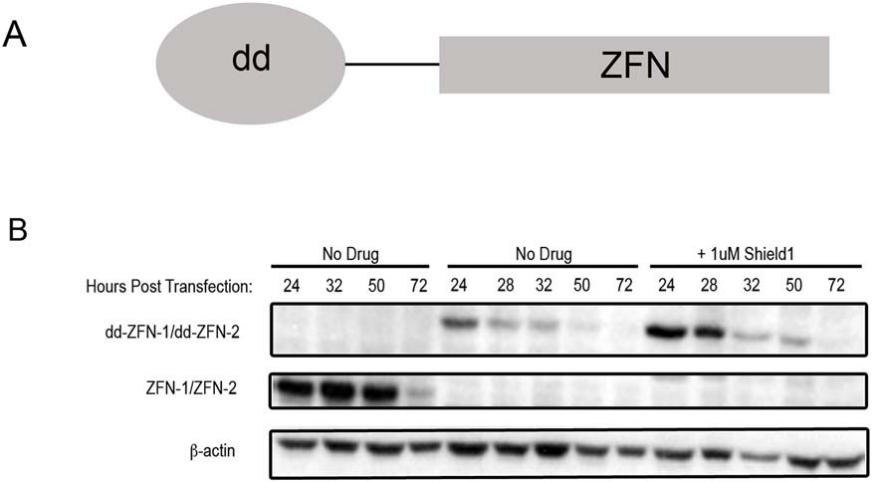
Characterization of dd-ZFNs that display Shield1-dependent stability. (A) Genetic fusion of a destabilization domain derived from an FKBP12 mutant to a ZFN. (B) Expression profile of unmodified and dd-ZFN proteins in the presence and absence of 1 uM of the Shield1 from 0–24 hours post-transfection. *HEK*293FT cells were transiently transfected with vectors encoding either ZFN-1/-2, dd-ZFN-1/-2. ZFNs were detected with an anti-Flag antibody. β-actin serves as a loading control. The molecular weight of the unmodified GFP-ZFNs was approximately 37 kD and for the dd-ZFNs approximately 50 kD.

We made a pair of chimeric proteins that linked the destabilization domain (dd) to the N-terminus of the ZFNs, containing the wildtype *FokI* domain, that target the GFP gene (“dd-ZFNs”, Figure 3A). We examined the expression of the dd-ZFNs and unmodified ZFNs in transfected HEK293 cells by Western blot analysis (Figure 3B). In the absence of Shield1, the dd-ZFNs were destabilized as shown by reduced expression relative to the unmodified ZFNs. Upon addition of Shield1 for the first 24 hours post transfection, however, the dd-ZFNs were stabilized to relatively equivalent levels of expression as the unmodified ZFNs at 24 hours. The amount of protein expressed at 32 hours post transfection after drug treatment, however, was substantially reduced when compared to the unmodified ZFNs at the same time point (Figure 3B).

**Figure 4:**
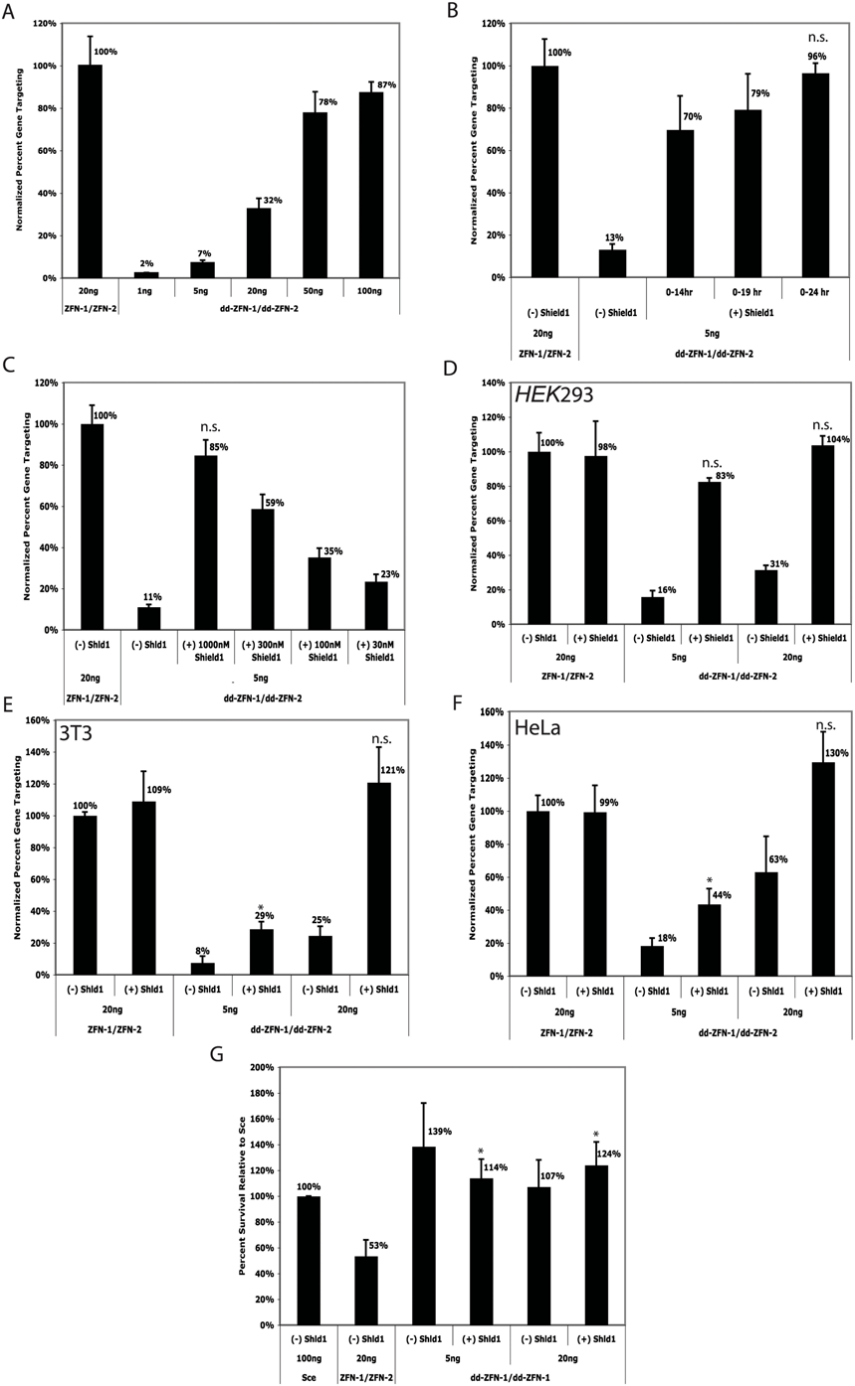
Analysis of dd-ZFNs. Unless otherwise indicated, rates of gene targeting at day 3 were normalized to the rate of gene targeting achieved using 20 ng of the unmodified ZFNs without drug treatment as this was previously determined to be the conditions used to obtain optimal gene targeting with the unmodified ZFNs. The absolute rate of gene targeting using ZFN-1/ZFN-2 at 20 ng in *HEK*293 cells was about 20,000 GFP positive cells per million cells transfected. (A) Titration of transfected DNA of dd-ZFNs in the gene targeting assay with increasing amounts of transfected DNA. (B) Time-course experiment for length of exposure of 1000 nM Shield1 using 5 ng of dd-ZFNs. Hours are given relative to the time of transfection, where “0” is the time of transfection. (C) Drug dose response curve for Shield1 with 5 ng of dd-ZFNs. (D) Gene targeting in the absence and presence of 1000 nM Shield1 for given ZFN pairs at stated DNA concentrations in *HEK293* cells. (E) Gene targeting in the absence and presence of 1000 nM Shield1 for given ZFN pairs at stated DNA concentrations in 3T3 cells. The absolute rate of gene targeting using ZFN-1/ZFN-2 at 20 ng in 3T3 cells was about 20,000 GFP positive cells per million cells transfected (2%). (F) Gene targeting in the absence and presence of 1000 nM Shield1 for given ZFN pairs at stated DNA concentrations in HeLa cells per million cells transfected. The absolute rate of gene targeting using ZFN-1/ZFN-2 at 20 ng in HeLa cells was about 2,000 GFP positive cells per million cells transfected (0.2%). (G) Toxicity assay for all iterations of dd-modified and unmodified ZFNs tested in the gene targeting assay relative to Sce. A value of <100% indicates decreased cell survival as compared with Sce, and demonstrates a toxic effect. Statistical analysis was performed using the Student's T-test comparing ZFN-1/ZFN-2 at 20 ng with no drug treatment to dd-modified ZFNs treated with Shield1. “*” indicates a P-value of <.05 and “n.s.” indicates no statistical significance or a P-value of >.05. Error bars are the standard deviation in measurement of three samples.

To examine the activity of the dd-ZFNs, we used the GFP gene targeting assay. At high concentrations of DNA, the rate of gene targeting stimulated by the dd-ZFNs in the absence of drug almost reached the rate stimulated by the unmodified ZFNs (Figure 4A). Because of this high rate of targeting in the absence of drug, we chose to continue the experiments with 5 or 20 nanograms of transfected DNA. We conducted a series of experiments to characterize the timing and dosing of the drug in order to determine the drug conditions needed to obtain optimal rates of gene targeting. After 24 hours of exposure to Shield1, the rate of gene targeting induced by the dd-ZFNs is equivalent to the rate stimulated by the unmodified ZFNs (Figure 4B). We found that additional exposure to the drug, beyond 24 hours, did not further increase these rates (data not shown).

We next evaluated the dosing of the Shield1 drug with respect to gene targeting. At 1000 nM of Shield1, we observed equivalent rates of gene targeting, but there was a dose-dependent decrease in targeting as the dose was lowered (Figure 4C). With Shield1 present at 1000 nM for the first 24 hours, we observed that using either 5 or 20 nanograms of the dd-ZFNs could produce rates of gene targeting equivalent to the optimal rates obtained with the unmodified ZFNs in HEK293 cells (Figure 4D). To determine if this method could be used in other cell types, we measured the gene targeting rates in HeLa and 3T3 cells stably transfected with the GFP gene targeting system. As shown in Figure 4E and 4F, the addition of Shield1 to cells transfected with the dd-ZFNs resulted in an increase in the rate of gene targeting relative to when the drug was absent. In the presence of Shield1, the rates of gene targeting in both the 3T3 cells and HeLa cells at 20 ng were equivalent to the rates produced using the unmodified ZFNs at 20 ng with no drug treatment (Figure 4E and F).

To determine if linking the destabilization domain to the ZFNs reduced the cytotoxicity associated with the unmodified ZFNs, we used the toxicity assay. Strikingly, in the presence of drug at 5 or 20 ng of dd-ZFNs, toxicity relative to Sce appears to be negligible, and there is a significant reduction in toxicity compared to the unmodified ZFNs at 20 ng (Figure 4G). We found that ZFNs with a N-terminal FKBP12 domain that is not destabilizing have greater toxicity than the dd-ZFNs, suggesting that the decreased toxicity is not simply the result of improved protein folding (data not shown).

**Figure 5:**
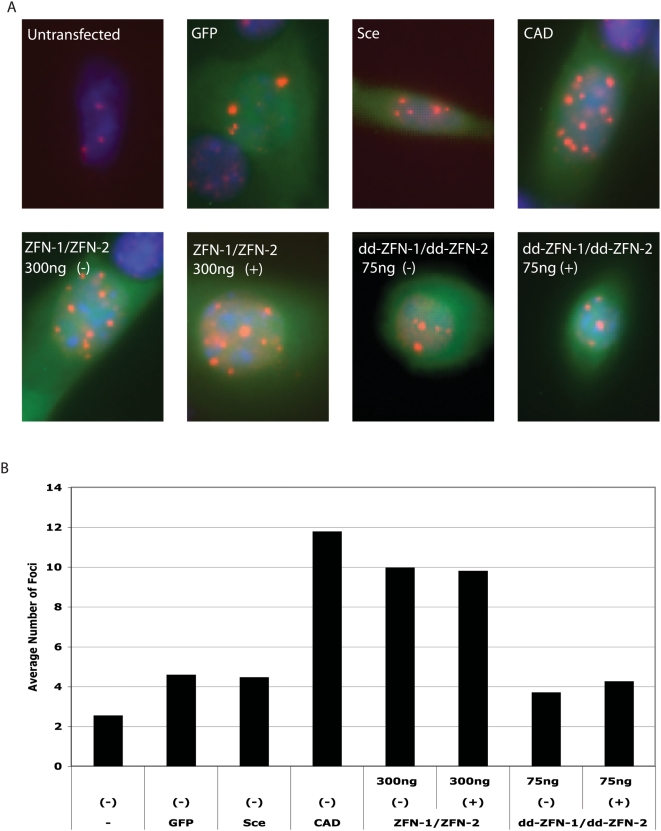
Visualization of ZFN-induced DSBs by sensitized 53BP1 foci formation assay. (A) Representative cells for each experimental condition after 53BP1 staining using indicated amounts of transfected DNA of each nuclease in the presence or absence of drug. 53BP1 foci are seen in red, 4,6-diamidino-2-phenylindole staining in blue, and GFP-positive cells in green. The foci were counted in transfected cells that were GFP-positive. “Untransfected” shows the background staining for foci in these cells, GFP indicates transfection of GFP alone, Sce serves as a negative control for ZFN-induced foci formation, and Caspase Activated DNAse (CAD) serves as a positive control for 53BP1 foci formation. ((−)) indicates no Shield1 treatment and ((+)) indicates 1000 nM Shield1 treatment for 24 hours after transfection. (B) The average number of 53BP1 foci per transfected cell in Ku80^−/−^ murine 3T3 cells for each experimental condition in (A).

Previous studies have suggested that the cytotoxicity associated with unmodified ZFNs is due to the creation of off-target DSBs [16]–[18]. When a DSB occurs, a signaling cascade is activated including the phosphorylation of H2AX and the recruitment of an array of proteins, including 53BP1, to the site of the DSB that can be detected as foci by immunofluorescence [16],[31]. We have previously shown that ZFNs that produce larger numbers of foci are more toxic than ZFNs that produce fewer foci [16]. Although the unmodified ZFN pair used in this study shows cytoxicity in the toxicity assay, this pair did not show an increased number of foci per cell relative to Sce when this assay was performed in human foreskin fibroblasts. To sensitize the assay, we used cells mutated in Ku80, a gene important in the non-homologous end-joining pathway of DSB repair, which are known to have delayed repair of DSBs [32]. In this cell line, GFP transfected cells and cells transfected with Sce alone had an average of about 4 foci per cell (Figure 5). As a further control, we transfected cells with a plasmid encoding Caspase Activated DNAse (CAD), an endonuclease that cleaves DNA non-specifically. CAD-transfected cells had an average of about 12 foci per cell (Figure 5). To aid in our comparison of the unmodified ZFNs and dd-ZFNs, we used higher amounts of DNA than determined in Figure 3 in order to amplify the number of DSBs visualized. We did however maintain the 1∶4 ratio (5 ng∶20 ng vs. 75 ng∶300 ng) of dd-ZFN DNA concentration with respect to unmodified ZFN DNA concentration for this comparison. Cells transfected with the unmodified ZFNs had an average of 10 foci per cell (comparable to the CAD transfected cells, Figure 5B). In contrast, the dd-ZFN transfected cells had only about 4 foci per cell (comparable to the GFP-alone and Sce transfected cells). In summary, linking the destabilization domain of a modified FKBP12 protein to the N-terminus of ZFNs resulted in a way to regulate the expression level of the ZFNs that maintained high rates of gene targeting while minimizing toxicity.

## Discussion

Homologous recombination is the most precise way to manipulate the genome and is a powerful experimental tool in several different systems. ZFNs have been shown to increase the rate of gene targeting in a wide variety of experimental systems previously not amenable to genome manipulation by homologous recombination [2],[10],[13],[17],[23],[24],[33]. In addition to the problem of designing ZFNs to recognize target sites [34], another limitation has been concern about off-target effects [2], [15]–[17]. Improvements in toxicity have been attained by increasing the specificity of ZFNs and by modifications of the nuclease domain [16], [19]–[24],[35]. Further strategies to minimize ZFN toxicity, however, could further broaden the window between the desired and undesired genomic effects of ZFNs. In whole organisms such as flies and zebrafish, high levels of ZFN expression led to abnormal developmental mutations [18],[24],[33]. Reducing ZFN toxicity by regulating ZFN expression could hypothetically help attenuate these abnormalities. In this work, we show that small molecule regulation of ZFN expression can result in an improved toxicity profile without sacrificing gene targeting activity. The use of the destabilization domain may not be necessary when making gene modified cell lines (where one can characterize a single clone) but instead will be useful when treating a large population of cells that may be infused into a patient (as would be done in gene therapy) where isolation of a single clone is either not feasible or desirable.

The standard strategy to control protein levels is to use transcriptional based methods (examples include the TetOn or TetOff: Clonetech, Ponasterone System; Stratagene, and Dimerizer System; Ariad). Gene targeting induced by ZFNs is already a three-component system (ZFN-1, ZFN-2, and a repair/donor molecule). Adding an inducible transcriptional regulator as a fourth component to make the system more complex was not desirable, particularly as the technology moves into cell types that are more difficult to transfect or infect. The ERT2 domain, a modified ligand binding domain from the estrogen receptor, has been successfully used to control protein activity by modulating the location of the protein. Unfortunately, we found that attaching the ERT2 domain to ZFNs did not stimulate gene targeting with presence of tamoxifen (data not shown). An alternative strategy is to use a post-translational method of regulating ZFN level. In this strategy, a destabilized ZFN is created by adding a destabilizing domain and then levels of ZFNs are controlled by adding a small molecule to block the destabilization effects. By fusing a ubiquitin domain to the N-terminus through a non-cleavable linker (Ub-VV-ZFN), we made ZFNs that could be regulated by proteasome inhibition, which resulted in decreased toxicity. When we fused the ubiquitin domain to the N-terminus of the ZFN through a cleavable linker leaving a destabilizing arginine at the N-terminus, we created ZFNs that were regulated by proteasome inhibition resulting in decreased toxicity and maintained high rates of gene targeting. Because proteasome inhibitors such as bortezomib are FDA approved for use in humans, this strategy has long-term promise. We did find the window of exposure to MG132, the proteasome inhibitor used in this study, in which we got good induction without cytotoxicity was narrow. Finally, when we fused a modified FKBP12 domain to the N-terminus of the ZFN, we created ZFNs that were regulated by the small molecule Shield1, which resulted in reduced ZFN toxicity and maintained high rates of targeting. Despite using amounts of Shield1 for prolonged periods (up to 48 hours), we did not observe any discernable toxicity. Moreover, by expression microarray analysis, Shield-1 has almost no effect on gene expression [36]. Thus, the Shield1/FKBP12 system may ultimately be the better system despite Shield1 not being currently FDA approved for use in humans.

Regulating ZFN expression also gave insight into the kinetics of gene targeting. Previously, Porteus and Baltimore found that maximal gene targeting was measured at 60 hours after transfection [2]. In this work, we demonstrate that ZFNs need only be expressed for less than 32 hours after transfection to attain maximal gene targeting (measured at 72 hours post-transfection). These experiments define a window for ZFN expression, here defined as 0–32 hours but perhaps even shorter, in which expression of ZFNs beyond the window does not increase targeting but does increase toxicity. We have no explanation for the 32-hour window for gene targeting based on experimental data. One would expect, for example, that gene targeting events should increase as long as ZFNs are present, but we do not observe this [2]. A hypothesis is that the repair substrate/donor may not be available (for example dilution, sequestration, or modification) for the repair of a double strand break by homologous recombination after this window. This hypothesis will have to be experimentally tested in the future.

Previously, we used human diploid fibroblasts to measure 53BP1 foci created by off-target DSBs. The unmodified ZFNs used in this study did not show significantly increased numbers of foci in that cell line, presumably because the cells were efficient at repairing DSBs. To sensitize the assay, we used murine Ku80^−/−^ cells that are deficient in DSB repair. Using these sensitized cells resulted in a higher number of background 53BP1 foci, but also allowed us to detect subtle differences in ZFN toxicity between dd-ZFNs and untagged ZFNs that we could not detect using cells that were not deficient in DSB repair. As ZFNs continue to improve, the use of sensitized assays to quantitate improvements will be an important strategy.

We have utilized strategies in which a drug stabilizes the protein rather than a drug to destabilize the protein. This “drug-on” system has several advantages. First, it means that the drug only needs to be administered for a brief period (the window to maximize gene targeting activity). This brief administration is advantageous because it is cheaper and because it minimizes the potential side-effects of the drug itself. Second, when the drug is absent, the ground state of ZFN expression will be low, thus reducing the potential side-effects of the ZFNs.

In summary, we have found that small molecule regulation of ZFN expression is an effective way to reduce cytotoxicity without compromising targeting efficiency. This strategy may be particularly beneficial to using ZFN mediated genome modification in a wide variety of cell types, including human stem cells.

## Materials and Methods

### DNA Manipulations and Cloning

All plasmids were made using standard cloning techniques and molecular biology as previously described [37]. The unmodified ZFNs were selected by the B2H design strategy and fused to the wildtype *FokI* nuclease domain as described earlier and called “GFP1.4-B2H” and “GFP2-B2H” [16]. For the Ub-X-ZFN versions, the ubiquitin open reading frame was amplified by PCR from pUb-R-GFP [28] with sense primer 5′-ACTGGGATCCTCTAGATCCACCATGCAGATCTTCGTGAAG-3′ and the antisense primers 5′-ACTGGGATCCAAGCTTCCC**CACCAC**ACCTCTGAGACGGAGTAC-3′ for the Ub-**VV**-ZFNs, or 5′-ACTGGGATCCAAGCTTCCC**TCT**GCCACCTCTGAGACGGAGTAC-3′ for the Ub-**R**-ZFNs (restriction site underlined, variable codons in bold) and cloned into the ZFN expression plasmid using the BamHI site. Directionality was determined by XbaI digest. To create the dd-ZFNs, the L106P destabilization domain was PCR amplified using primers 5′-ACGTGCGGCCGCACCATGGGAGTGCAGGTGGAAACCATCTCC – 3′ and 5′-ACTGGGATCCTTCCGGTTTTAGAAGCTCCAC-3′. The resulting fragment was digested with NotI and BamHI and cloned in-frame to the N-terminus of the GFP-ZFNs in a CMV expression vector. For all constructs the N-terminal domains and junctions were confirmed by sequencing.

### Cell Culture and Transfection

All cell culture experiments were performed in *HEK*293 cells except where identified. Cells were cultured in a humidified incubator at 37°C with 5% CO_2_ in DMEM supplemented with 10% bovine growth serum (Hyclone, Logan, UT, USA), 2 mM L-glutamine, 100 IU/ml penicillin, and 100 mg/ml streptomycin. Stable cell lines were constructed as previously described [15]. Transient transfections were performed using the calcium phosphate technique as previously described and produced transfection efficiencies between 10–35% [38].

### Proteasome Inhibitor

For experiments using MG132 (carboxybenzyl-leucyl-leucyl-leucinal; Sigma-Aldrich, St. Louis, MO),10 uM drug was added to cells from 18–22 hours post-transfection unless otherwise noted. We determined this window and concentration of the proteasome inhibitor empirically to maximize stimulation of gene targeting while minimizing toxic effects of the drug (data not shown).

### Shield1

For experiments using Shield1 (Clontech, Mountain View, CA), 1000 nM drug was added to cells from time of transfection and left on for 24 hours unless otherwise noted. As discussed in the results, we determined the dose and timing of the drug empirically to maximize gene targeting activity and minimizing toxicity.

### Measurement of Gene Targeting using the GFP System in HEK293 Cells

Gene targeting experiments were performed in triplicate as previously described using calcium phosphate transfection [15]. Transfection efficiencies were determined at day 2 post-transfection, and the rates of gene targeting were determined by flow-cytometry and analyzed on a FACS Calibur (Becton-Dickinson, San Jose, CA, USA) at day 3 (day of transfection is considered day 0). Gene targeting rates are calculated as GFP positive cells per million cells transfected because the background rate of spontaneous gene targeting using this system is approximately one event per million cells. Gene targeting rates are then normalized to the percent gene targeting obtained using 20 ng of ZFN-1 and ZFN-2 as these conditions have given the highest rates of gene targeting for the unmodified proteins. The absolute rate of gene targeting using ZFN-1/ZFN-2 at 20 ng in HEK293 cells was about 20,000 GFP positive cells per million cells transfected.

### Measurement of Gene Targeting using the GFP System in HeLa and 3T3 Cells

Both a HeLa and 3T3 cell line were created using electroporation that stably incorporated the GFP gene targeting system. Gene targeting experiments were performed in triplicate as previously described [15]. Lipofectamine 2000 Reagent (Invitrogen) was used to transfect cells using Invitrogen's suggested protocol. pcDNA6/V5-HisA plasmid DNA was added as stuffer DNA when necessary to raise the total DNA to 800 ng per well. 1000 nM Shield1 was added to drug-treated wells at the time of transfection. 24 hours later, Shield1 was removed and the medium was replaced with fresh, supplemented Dulbecco's Modified Eagle's Medium. The absolute rate of gene targeting using ZFN-1/ZFN-2 at 20 ng in HeLa cells stably transfected with GFP gene targeting reporter was about 2,000. The absolute rate of gene targeting using ZFN-1/ZFN-2 at 20 ng in 3T3 cells stably transfected with the GFP gene targeting reporter was about 20,000.

### Immunodetection of ZFNs

For time course blots, cells were harvested at indicated times post-transfection. Each sample was counted and lysate volumes were adjusted to give equal amounts of cells per volume. Equal amounts of total lysates were subjected to SDS-PAGE, wet transferred to PVDF membranes and incubated with specific antibodies. ZFNs were detected using an anti-Flag M2 monoclonal antibody (1∶10,000, Sigma-Aldrich), and β-actin was detected using a rabbit anti-actin antibody (1∶5,000, Sigma-Aldrich). The blots were further incubated with HRP-conjugated secondary antibodies and visualized using Western blotting luminal reagent (Santa Cruz Biotechnology, Santa Cruz, CA).

### Flow Cytometry-Based Assay for Cell survival: “Toxicity Assay”

Toxicity assays were performed as previously described [16]. Briefly, *HEK*293 cells were transfected in triplicate by calcium phosphate technique with 200 ng of a GFP expression plasmid and with varying amounts of each nuclease expression plasmid (two plasmids total). At day two post-transfection, a fraction of transfected cells was analyzed by flow-cytometry and the percentage of GFP positive cells was determined. At day six post-transfection, the percentage of GFP positive cells was determined by flow-cytometry. To calculate the percent survival relative to Sce, a ratio of ratios was calculated as previously described [16]. The ratio after nuclease transfection was normalized to the ratio after Sce transfection and this determined the percent survival compared to Sce. In control experiments, we showed that Sce expression had no effect on cell survival compared to cells transfected with an empty expression vector.

### Sensitized 53BP1 Foci Formation Assay

Cell Culture: Ku80^−/−^ mouse 3T3 cells were maintained in Dulbecco's Modified Eagle's Medium (Hyclone, Logan, UT) supplemented with 20% fetal calf serum and 2 mmol/l L-glutamine. The cells were maintained in a humidified incubator with 5% CO_2_ at 37°C.

Transfection of 3T3 Ku80^−/−^ cell line: Mouse 3T3 cells that are Ku80^−/−^ were used in these studies because they repair DNA breaks more slowly, providing a more sensitive assay for monitoring DNA damage. Ku80^−/−^ cells were seeded in 4-well Lab-Tek II Chamber Slides (Nalge Nunc, Rochester, NY) at 40,000 cells per well. 24 hours later, cells in each well were lipofected with 200 ng GFP DNA and 75 ng or 300 ng of each nuclease. pcDNA6/V5-HisA plasmid DNA was added as stuffer DNA when necessary to raise the total DNA to 800 ng per well. Lipofectamine 2000 Reagent (Invitrogen) was used to transfect cells using Invitrogen's suggested protocol. 1000 nM Shield1 was added to drug-treated wells at the time of transfection. 24 hours later, Shield1 was removed and the medium was replaced with fresh, supplemented Dulbecco's Modified Eagle's Medium. 48 hours after lipofection the cells were fixed, stained and visualized. 53BP1 foci were counted only in cells that were brightly GFP positive because these were the ones transfected with the GFP and the nuclease(s).

### Immunofluorescence Staining

Immunofluorescence staining was carried out as performed in [16]. Briefly, cells were washed in phosphate buffered saline, fixed in cold 4% paraformaldehyde, washed again, and then permeabilized with .5% Triton X-100. Cells were re-washed, blocked in 5% bovine serum albumin, and then incubated with rabbit anti-53BP1 antibody (Cell Signaling, Danvers, MA). After another set of washes, cells were incubated with goat anti-rabbit Rhodamine Red-X antibody (Invitrogen, Carlsbad, CA). Cells were washed again and then mounted in Vectashield mounting medium containing 4,6-diamidino-2-phenylinodole (Vector Laboratories, Burlingame, CA). Images were captures using an epifluorescence microscope equipped with a Q-Fire charge-coupled device camera (Olympus America, Melville, NY) and QCapture Software (QImaging, British Columbia, Canada). Images were merged using ImageJ Software (NIH, ver. 1.40 g).
